# Draft Genome Sequences of Two Clinical Methicillin-Resistant *Staphylococcus aureus* Strains Isolated from Healthy Children in Brazil

**DOI:** 10.1128/genomeA.00158-17

**Published:** 2017-04-13

**Authors:** Suzi P. de Carvalho, Jéssica B. de Almeida, Leandro M. de Freitas, Ana Marcia S. Guimaraes, Naíla C. do Nascimento, Andrea P. dos Santos, Joanne B. Messick, Jorge Timenetsky, Lucas M. Marques

**Affiliations:** a State University of Santa Cruz (UESC), Campus Soane Nazaré de Andrade, Ilhéus, Brazil; b Multidisciplinary Institute of Health, Universidade Federal da Bahia, Vitória da Conquista, Brazil; c Department of Microbiology, Institute of Biomedical Science, University of São Paulo, São Paulo, Brazil; d Department of Comparative Pathobiology, Purdue University, West Lafayette, Indiana, USA

## Abstract

We report here the draft genome sequences of two community-associated methicillin-resistant *Staphylococcus aureus* (CA-MRSA) strains, C18 and C80, isolated from healthy children from day care centers. To our knowledge, these are the first draft genome sequences of CA-MRSA ST398/CC398/Scc*mec*V and CA-MRSA ST5/CC5/Scc*mec*IVa isolated from healthy children in Brazil.

## GENOME ANNOUNCEMENT

Community-associated methicillin-resistant *Staphylococcus aureus* (CA-MRSA) is considered pandemic and has contributed significantly to the rise of a wide range of clinical diseases associated with staphylococcal infections (1). Accordingly, the incidence of pediatric infections due to CA-MRSA, including in healthy children, has increased worldwide (2). We present here the genome sequences of two CA-MRSA isolates from nasal swabs of healthy children attending public day care centers in Vitória da Conquista, Bahia, Brazil.

Two *S. aureus* strains (identified as C18 and C80) isolated from healthy children and identified as MRSA using a microdilution method and PCR were genotyped by multilocus sequence typing, pulsed-field gel electrophoresis, and *spa* typing and by the presence of staphylococcal chromosomal cassette *mec* (SCC*mec*) and virulence genes. Strain C18 was identified as sequence type 398 (ST398), clonal complex 398 (CC398), *spa* type t242, SCC*mec* type V, and as a carrier of *eta*, *seg*, *sei*, and *icaD*. Strain C80 was classified as ST5, CC5, t002, SCC*mec* Iva, and as a carrier of *seg*, *sei*, and *icaD*. Invasive infection by CC398 rarely occurs (3), but strain CC5 belongs to a clonal group known to be involved in a global pandemic caused by MRSA (4). Genomic DNA was extracted using a PureLink Genomic DNA minikit (Life Technologies, Inc., Brazil). The whole genome was sequenced from a paired-end library using the Illumina HiSeq 2500 platform (Illumina, Inc., San Diego, CA, USA) at the Purdue University Genomics Core Facility. Average reads of about 100 bp were assembled using ABySS version 1.2.7. After assembly resulting from 1,256× (strain C18) and from 2,060× (strain C80) genomes coverages, first-pass annotation was achieved using the NCBI Prokaryotic Genome Annotation Pipeline.

A total of 36,012,182 reads of strain C18 were assembled into 123 contigs, having a genome size of ~2,630 Mb with a G+C content of 33.6%. First-pass annotation of strain C18 strain resulted in 2,575 genes, 2,481 coding DNA sequences (CDSs), 43 RNA-coding genes (34 tRNAs, five rRNAs, and four ncRNAs), and 94 pseudogenes. The resistome for strain C18 was analyzed using ResFinder version 2.1 (https://cge.cbs.dtu.dk//services/ResFinder), and the following resistance genes were identified: *spc* (aminoglycoside resistance), *mecA* (beta-lactam resistance), *blaZ* (beta-lactam resistance), and *erm*_*(A)*_ (macrolide resistance).

Regarding strain C80, a total of 48,981,312 reads were assembled into 36 contigs. The draft genome sequence of *S. aureus* C80 has a size of ~2,692 Mb with a G+C content of 32.8%. A total of 2,737 genes, 2,670 CDSs, 48 RNA-coding genes (39 tRNAs, five rRNAs, and four ncRNAs), and 67 pseudogenes were annotated. In the resistome of strain C80, the following resistance genes were identified: *norA* (fluoroquinolone resistance), *mecA* (beta-lactam resistance), and *blaZ* (beta-lactam resistance).

To our knowledge, these are the first draft genome sequences of CA-MRSA ST398/ CC398/Scc*mec*V and CA-MRSA ST5/CC5/Scc*mec*IVa isolated from healthy children in Brazil. Further genomic analyses of CA-MRSA strains isolated from healthy people in Brazil are required in order to understand the molecular characteristics involved in the *S. aureus* pathogenicity and to elucidate epidemiological aspects regarding the dissemination of antibiotic resistance genes among healthy children.

### Accession number(s).

The genome sequences of methicillin-resistant clinical *Staphylococcus aureus* strains C18 and C80 have been deposited in GenBank under the accession numbers MSFF00000000 and MSFE00000000, respectively.
